# Metabolomic and lipidomic insights into the impact of *Euglena gracilis*-derived β-glucan supplementation on sow colostrum and milk composition

**DOI:** 10.1016/j.csbj.2025.02.033

**Published:** 2025-02-27

**Authors:** Jakavat Ruampatana, Takele Feyera, Unchean Yamsrikaew, Methaporn Juarjan, Kunaporn Homyog, Wanwimon Mekboonsonglarp, Sarn Settachaimongkon, Morakot Nuntapaitoon

**Affiliations:** a Department of Obstetrics, Gynaecology and Reproduction, Faculty of Veterinary Science, Chulalongkorn University, Bangkok 10330, Thailand; b Department of Animal Veterinary Sciences, Aarhus University, AU-Viborg, Tjele, DK 8830, Denmark; c Department of Food Technology, Faculty of Science, Chulalongkorn University, Bangkok 10330, Thailand; d Center of Veterinary Diagnosis, Faculty of Veterinary Science, Mahidol University, Nakhon Pathom 73170, Thailand; e Scientific and Technological Research Equipment Center (STREC), Chulalongkorn University, Bangkok 10330, Thailand; f Omics Sciences and Bioinformatics Center, Faculty of Science, Chulalongkorn University, Bangkok 10330, Thailand; g Center of Excellence in Swine Reproduction, Chulalongkorn University, Bangkok 10330, Thailand

**Keywords:** Colostrum, *Euglena gracilis*, Lipidomics, Metabolomics, Prebiotic

## Abstract

*Euglena gracilis,* an algae-derived source of β-glucan, exhibits prebiotic activity that enhances colostrum quality and improves growth, though the underlying mechanisms remain unclear. This study investigates the effects of *E. gracilis* supplementation during late gestation and lactation on sow colostrum and milk biomolecular profiles. Sixty-one crossbred sows (Landrace × Yorkshire) were assigned to a standard diet (CON; *n* = 30) or the CON diet supplemented with 1 g/sow/day of *E. gracilis* (TRT; *n* = 31) from day 85 of gestation until day 21 of lactation. Sow performance, including litter size and weight, was recorded from birth to day 21 of age. Colostrum samples (*n* = 20; 10 sows/group) were collected within 1 h of farrowing, and milk samples were collected from the same sows on days 3 and 10 of lactation to assess alterations in non-volatile polar metabolites (NVM), fatty acids (FA), and associated metabolic pathways. On average, the litter size at birth was 14.2 ± 2.5 piglets/litter, with no effect of dietary treatment from birth to day 21 of lactation (*P* > 0.05). However, piglets suckled by TRT sows tended to have higher average daily gain from birth to day 21 of age than those suckled CON sows (191.0 ± 6.7 vs. 173.6 ± 6.8 g/day, *P* = 0.073). Chemometric analysis revealed distinct NVM and FA profiles between the groups, particularly in the colostrum. Although *E. gracilis* supplementation influenced the contents of multiple metabolites, focus has been given to those that have direct impact on piglet development, including increased colostrum leucine (*P* = 0.001), threonine (*P* < 0.001), and N-acetylglucosamine (*P* = 0.002), enhancing colostrum quality and immunomodulatory potential. Elevated colostrum gamma-linolenic acid (*P* = 0.047) and arachidonic acid (*P* = 0.019) levels suggested enhanced immune development. Pathways associated with amino sugars and nucleotide sugars and glucose-related metabolism in colostrum were also modulated. These findings suggest *E. gracilis*-derived β-glucan as a potential dietary supplement for enhancing sow colostrum quality and piglet growth.

## Highlights

• *E. gracilis* modulated sow colostrum and milk metabolite and fatty acid profiles, tending to improve piglet growth.
• Metabolomic analyses identified potential biomarkers in colostrum linked to improved piglet growth.
• Increased colostrum leucine and GlcNAc levels were associated with higher piglet growth.
• Increased colostrum threonine, gamma-linolenic acid, and arachidonic acid levels suggested immunomodulatory potential.

## Graphical Abstract

**Figure:**
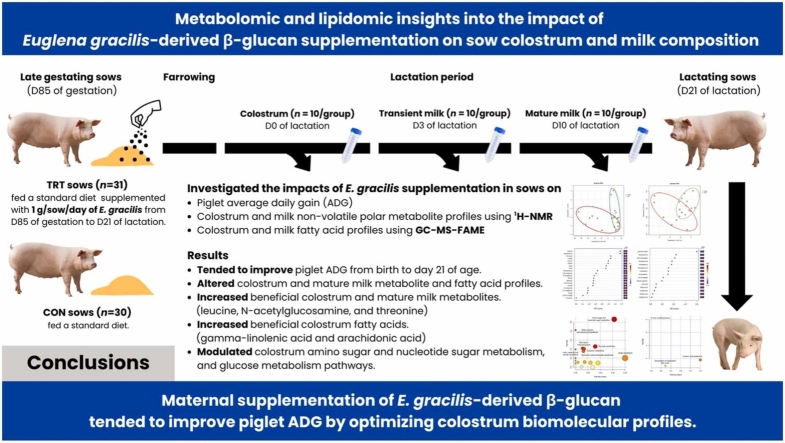

## 1 Introduction

The neonatal period is critical for piglet survival and growth, necessitating optimal intake of colostrum to enhance the likelihood of weaning. Colostrum, a vital source of nutrients and immunoglobulin (Ig), plays a key role in sustaining energy homeostasis and supporting development of newborn piglets [1], [2]. Colostrum nutrients and IgG are essential for promoting growth and providing resistance against infection, respectively [1], [3]. Furthermore, biomolecular compounds in colostrum and milk, such as essential and non-essential amino acids (AA), can contribute to piglet development and overall health [3], [4]. Consequently, exploring strategies to improve colostrum quantity and quality, and thereby optimizing piglet performance, has been a topic of research interest for decades.

β-glucan, a prebiotic found in the cell walls of bacteria, fungi, algae, yeast, and cereal grains [5], exhibits functionality that depends on the physicochemical properties of its source, influencing its bioavailability, bioactivity, and efficacy [6]. Among these sources, *Euglena gracilis*, an algae-derived β-glucan, has gained attention for its immunomodulatory effects on sow colostrum, thereby improving piglet growth [7]. However, other beneficial biomolecular compounds in colostrum and milk that play acrucial role in piglet performance may also be affected by *E. gracilis* supplementation [3], [4]. Therefore, investigating how *E. gracilis* supplementation affects the biomolecular profiles of sow colostrum and milk could provide insight into its positive effects on piglet performance, as its underlying mechanism remains unclear.

Metabolomics is a useful tool for identifying and quantifying small molecular weight metabolites, providing insights into biochemical changes in milk composition [8]. This approach utilizes advanced analytical instruments, such as mass spectrometry and nuclear magnetic resonance spectroscopy, enabling high-throughput and comprehensive metabolite profiling to determine if molecular metabolites are influenced by factors such as lactation stage, breed, parity, health status, and dietary supplementation [9], [10], [11], [12], [13]. While extensively applied in dairy research, the use of metabolomics in swine colostrum and milk studies, particularly in response to probiotic supplementation, remains limited. Therefore, this study aimed to investigate the impact of *E. gracilis* supplementation during late gestation and lactation periods on the alterations in non-volatile polar metabolite (NVM) and fatty acid (FA) profiles of sow colostrum and milk. It was hypothesized that maternal *E. gracilis* supplementation would optimize mammary gland secretions and enhance piglet growth.

## 2 Materials and methods

The use of animals in this experiment was approved by the Institutional Animal Care and Use Committee (IACUC) at Chulalongkorn University (Approval number: 2331062).

### 2.1 Experimental design and animal feeding

The experiment was conducted on a commercial farm with a capacity of 2000 sows in the central region of Thailand from July to August 2023. A total of 61 Landrace × Yorkshire crossbred sows with a mean parity number of 2.8 ± 1.7 (ranging from 1 to 7) were included in this study from late gestation until day 21 of lactation. The average lactation period on the farm was 31.9 ± 1.7 days; however, data collection for this experiment concluded on day 21 of lactation. Throughout the experiment, housing temperature was monitored using a Temperature Data Logger (EasyLog-USB-1-LCD, LASCAR electronic, Hong Kong SAR, PRC), with an average temperature of 28.4 ± 1.7 ºC (ranging from 25.0 ºC to 34.5 ºC). During gestation, sows were housed individually in stalls (2.2 m × 0.8 m) within a gestation house equipped with an evaporative cooling system. During gestation, the sows were fed twice daily with a gestation diet at 07:30 a.m. and 01:30 p.m., with an average daily supply of 3.0 kg/day. The standard gestation diet was formulated to meet or exceed the nutritional requirements outlined by NRC [14], and its nutrient composition is presented in Table 1**.** On day 85 of gestation, the sows were stratified for parity into three classes (parity 1, 2–4, and 5–7) and assigned to the standard gestation diet (CON; *n* = 30) or the CON diet supplemented with 1 g/sow/day of *E. gracilis*-derived β-glucan (Aleta™) as top dressing during the morning meal (TRT, *n* = 31). The sows were fed the dietary supplement from day 85 of gestation until day 21 of lactation (54.5 ± 4.7 days). *E. gracilis*-derived β-glucan (Aleta™) used in this experiment was provided by Kemin Industries (Asia) Pte Ltd, SG., and the product is expected to contain 50 % β-(1,3) glucan according to the producer information provided by the supplier. Furthermore, the details of nutrient ingredients and levels of the standard gestation diet and *E. gracilis*-derived β-glucan supplement are presented in Table 1. Sows had *ad libitum* access to water through drinking nipples throughout gestation.

**Table 1:** Nutrient composition of the gestation diet, lactation diets, and *E. gracilis*-derived β-glucan (as-fed basis).

Item	Gestation diet	Lactation diet	*E. gracilis*
Nutrient ingredients (g/kg)			
Corn	323	472	
Cassava	199	74.6	
Broken rice	323	149	
Soybean meal	104	244	
Soybean oil	7.46	32.3	
Poultry meal		22.4	
Dicalcium phosphate	12.4	14.9	
Limestone	17.4	14.9	
Salt	3.98	3.98	
Lysine	1.49	2.74	
Methionine	0.52	0.25	
Threonine	1.00	1.00	
Mycotoxin binder	1.00	1.00	
Premix^1^	1.00	1.00	
Nutrient compositions (%)			
ME (kcal/kg)	2783	3363	3799
Crude protein	11.8	16.2	16.1
Crude fat	5.73	8.39	5.78
Crude fiber	4.63	4.70	1.73
Ash	11.3	7.53	4.41
Moisture	9.72	9.97	1.29
Nitrogen free extract			70.7

1 Supplied per kg of diet: vitamin A 16 000 IU, vitamin D3 3 250 IU, vitamin E 60 IU, vitamin K3 2 mg, vitamin B1 4 mg, vitamin B2 8 mg, vitamin B3 6 mg, vitamin B12 0.03 mg, vitamin C 0.15 mg, pantothenic acid 16 mg, niacin 30 mg, folic acid 2 mg, biotin 0.35 mg, choline 450 mg, Mn 60 mg, Fe 120 mg, Zn 125 mg, Co 2 mg, Cu 12 mg, I 0.25 mg, Se 0.2 mg, Mg 65 mg.

The sows were moved from the gestation house to the farrowing unit 7 days before the expected farrowing date and then kept in the farrowing pens (2.2 m × 2.0 m) equipped with an evaporative cooling system until weaning on day 31.9 ± 1.7 of lactation. Each pen included a creep area for the piglets (0.8 m × 0.5 m) with a heating lamp and rubber mattress. At the farrowing house, all sows received the standard lactation diet, with its nutrient ingredients and levels detailed in Table 1. This diet was provided twice daily at 07:30 a.m. and 01:30 p.m. The feeding level gradually increased by 0.5 kg/day from farrowing until day 7 of lactation until it reached the maximum daily allowance of 6.0 kg, which was then mentained during the rest of the lactation period. The farrowing process was monitored, and farrowing assistance was provided when the birth interval exceeded 45 minutes or if uterine contractions stalled. After the birth of the eighth piglet, sows were administered oxytocin (10 IU/mL, VetOne®, Idaho, USA) intravenously to modulate fetal expulsion. Water was provided *ad libitum* to both sows and piglets during the lactation period.

### 2.2 Data collection

Piglet performance data, including litter weight and size at birth, and on days 3, 10, and 21 of age, were recorded. Litter weights were measured using a digital balance (B6S Weighing Indicator, ZEPPER Instrument Co., Ltd., Nonthaburi, Thailand). Average daily gain (ADG) of the piglet in each litter was calculated by dividing the difference in litter weight between measurement days by the number of days and the litter size.

### 2.3 Samples collection

A total of 20 sows, consisting of 10 sows per group and 5 sows per parity class, were selected for colostrum and milk sampling. Colostrum samples (25 mL) were manually collected from all functional teats within 1 h after the onset of farrowing. Milk samples (25 mL) were also collected from the same sows on days 3 (transient milk) and 10 (mature milk) of lactation after a 0.1 mL intravenous injection of oxytocin (10 IU/mL, VetOne®, Idaho, USA). All samples were filtered through gauze, transferred to clean 30 mL bottles, and stored in a Styrofoam box during sampling before being frozen at −20 °C for further analysis.

### 2.4 Determination of non-volatile polar metabolite profiles in sow colostrum and milk

Non-volatile polar metabolites in sow colostrum and milk were analyzed using a non-targeted proton nuclear magnetic resonance (^1^H NMR)-based metabolomics approach, according to the method used in the previous study [9], [15]. Briefly, the sample pH was adjusted to 6.0, and lipid and large protein fractions were removed via dichloromethane extraction and ultracentrifugation (74200 × g for 60 min at 4 °C), respectively. The supernatant was then filtered through a Pall Nanosep® centrifugal device with 3 kDa molecular weight cutoffs (Pall Life Sciences, Michigan, USA). Finally, the clear milk serum was mixed 1:1 (v/v) with phosphate buffer (pH 6.0) containing 1 mM 3-(Trimethylsilyl) propionic-2, 2, 3, 3-d4 acid sodium salt (Merck, Darmstadt, Germany) as an internal standard. The samples were analyzed using a 600 MHz NOESY-GPPR-1D-^1^H NMR spectrometer (Bruker, Rheinstetten, Germany) operated with similar parameters as described in the previous study [9]. ^1^H NMR spectra were corrected, pre-treated, and segmented using the binning technique. Metabolite identification was performed by consulting the Chenomx NMR suite 8.2 library (Chenomx Inc., Canada), Livestock Metabolome Database (www.lmdb.ca), and literature sources [9], [10], [11], [12], [13]. The sum of signal intensities corresponding to respective metabolites was expressed in arbitrary units and further introduced as variables in statistical analysis.

### 2.5 Determination of fatty acid profiles in sow colostrum and milk

Fatty acid compositions of colostrum and milk samples were determined using gas chromatography coupled with mass spectrometry for fatty acid methyl ester (GC-MS-FAME) analysis (Agilent 7890A-5975C, Agilent Technologies, Santa Clara, CA, USA) according to the method described in the previous study [9]. Briefly, fatty acid methyl ester (FAME) formation was initialized after heating and hydrolysis of samples with KOH, MeOH, and H_2_SO_4_. The FAME fractions were collected after hexane extraction. The FA profiles of the FAME fraction were determined by capillary GC on a SP-2560 capillary column (Supelco, Bellefonte, PA, USA) operated using similar parameters as described previously [9]. The FA were identified by comparing their specific retention time and *m/z* model with a FAME standard (Supelco 37 Component FAME mix, Sigma-Aldrich, Steinheim, Germany). After automated peak integration, the concentrations of FA were calculated using calibration curves fitted by a linear regression model and finally expressed as mg/100 g [9].

### 2.6 Statistical analyses

All statistical analyses were conducted using SAS version 9.0 (SAS Institute Inc., Cary, NC, USA). Data normality was assessed using the UNIVARIATE procedure, and no transformations were required to stabilize residual variance. Descriptive statistics for the continuous variable was analyzed using MEANS procedure of the SAS. The effects of dietary supplementation (CON and TRT) on litter size and piglet ADG were analyzed using the general linear model procedure of the SAS. Additionally, the effects of dietary supplementation at different lactation stages (colostrum, transient milk, and mature milk) on NVM and FA profiles were assessed using the general linear mixed procedure. The statistical model applied was:

Ƴ_i_ = μ + α_i_ + δ_j_+ Ɛ_k_

Where Ƴi is the response variable, μ is the overall mean, α_i_ is the fixed effect of dietary supplement (i = CON and TRT), δ_j_ is the random effect of sow within CON and TRT groups, and Ɛ_k_ is the residual error. Least-square means were calculated and compared using the Tukey-Kramer test. For all analyses, a *P*-value < 0.05 was considered statistically significant, while a *P*-value < 0.10 was considered as a tendency.

^1^H NMR-derived metabolomic and GC-derived lipidomic data were analyzed and compared using multivariate statistical analysis in MetaboAnalyst 5.0 (www.metaboanalyst.ca). Partial least squares discriminant analysis (PLS-DA) was applied to visualize distinct patterns in NVM and FA profiles between groups and variable importance in projection (VIP) scores [9]. The quality of the PLS-DA model was assessed by the leave-one-out cross validation method and expressed by the prediction accuracy (%), *R*^2^ (accuracy), and *Q*^2^ (predictability). The compounds with VIP scores > 1.0 and *P*-values < 0.05 were considered potential biomarkers responsible for discrimination. The *in silico* pathway analysis was performed to detect differential metabolite and FA biosynthesis based on the pathway library of *Sus scrofa* in Kyoto Encyclopedia of Genes and Genomes database.

## 3 Results

### 3.1 Piglet performance

**Fig. 1:**
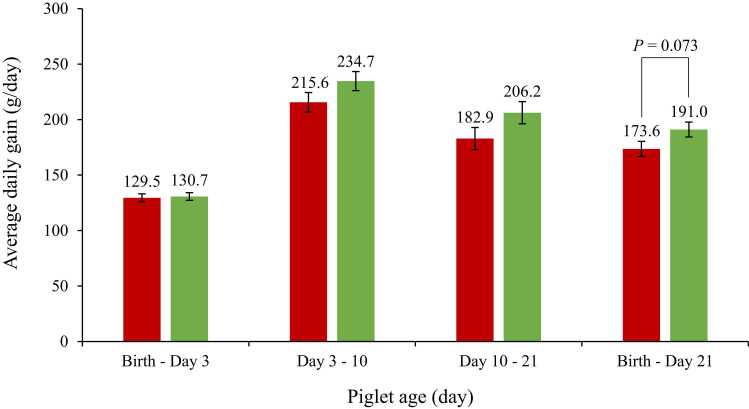
Average daily gain from piglets suckled by sows fed a standard gestation diet from day 85–108 of gestation and standard lactation diet from day 109 of gestation until day 21 of lactation (**CON**; white color) or fed the CON diet top dressed with 1 g per sow per day of *E. gracilis*-derived β-glucan (**TRT**; black color) (Least square mean ± SEM).

The average litter size across all sows in this study was 14.2 ± 2.5 piglets/litter at birth and 11.9 ± 1.9 piglets/litter on day 21 of lactation, with no differences between CON and TRT sows from birth to day 21 (*P* > 0.05; Table 2). However, piglets suckled by TRT sows tended to have a higher ADG from birth to day 21 of age than those suckled by CON sows (*P* = 0.073; Fig. 1). No differences were observed during the interval between birth to day 3 (*P* = 0.803), day 3–10 (*P* = 0.147), and day 10 to day 21 of age (*P* = 0.107).

**Table 2:** Average litter size in sows fed a standard gestation diet from day 85–108 of gestation and standard lactation diet from day 109 of gestation until day 21 of lactation (CON) or fed the CON diet top dressed with 1 g/sow/day of *E. gracilis*-derived β-glucan (TRT) (Least square mean ± SEM).).

Item	CON	TRT	SEM^1^	*P*-value
Number of sows	30	31		
Litter size (piglets/litter)				
At birth	14.2	14.3	0.465	0.929
Day 1 of lactation	13.2	13.2	0.405	0.964
Day 3 of lactation	12.8	12.9	0.310	0.871
Day 10 of lactation	12.0	12.2	0.311	0.659
Day 21 of lactation	11.9	11.8	0.349	0.746

1 Greatest standard error of the mean (SEM).

### 3.2 Non-volatile polar metabolite profiles of sow colostrum and milk

**Fig. 2:**
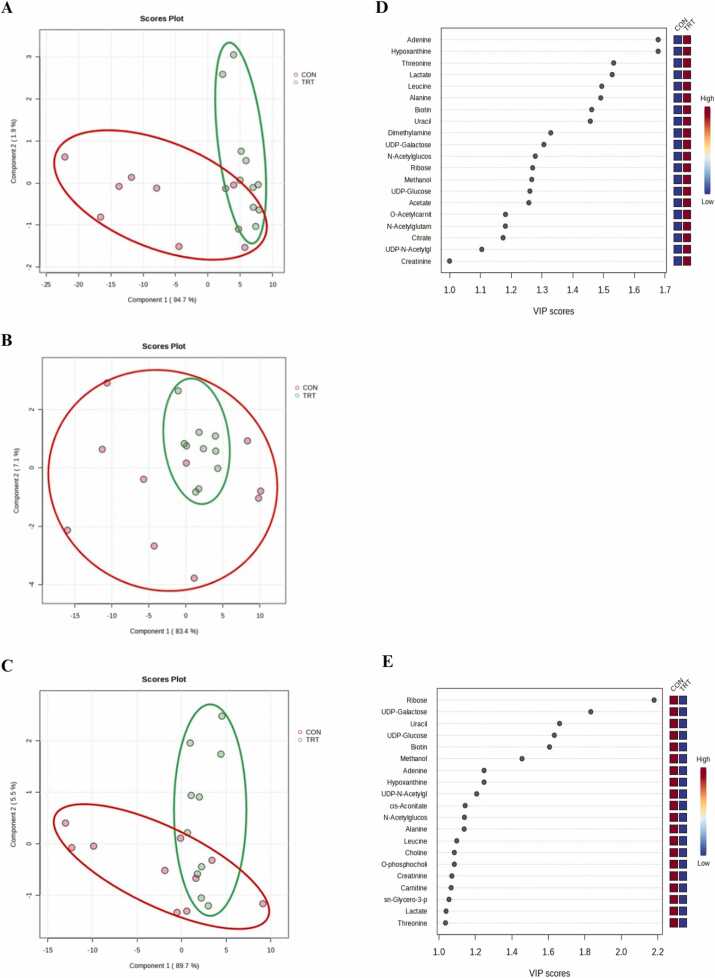
PLS-DA scores plot for the comparison of metabolite profiles of colostrum (panel **A**), transient milk (panel **B**), and mature milk (panel **C**) samples collected from sows fed a standard gestation diet from day 85–108 of gestation and standard lactation diet from day 109 of gestation until day 21 of lactation (**CON**; red color) or fed the CON diet top dressed with 1 g/sow/day of *E. gracilis*-derived β-glucan (**TRT**; green color). PLS-DA derived VIP scores derived from the comparison among samples of the same day postpartum and indicative potential biomarkers for discrimination in colostrum (panel **D**) and mature milk (panel **E**).

A total of 45 non-volatile polar metabolites, including alcohols and polyols, amines, AA and derivatives, carbohydrates and derivatives, and organic acids, were identified in sow colostrum, transient milk, and mature milk (Table S1). The PLS-DA score plots were generated separately for colostrum, transient milk, and mature milk and presented in Fig. 2. In colostrum, PLS-DA scores plot revealed a clear distinction between groups, with a prediction accuracy of 90.00 %, *R²* = 0.793, and *Q²* = 0.655 (Fig. 2**A**). No distinct pattern was observed in transient milk (Fig. 2**B**). However, mature milk displayed a clear separation, with a prediction accuracy of 75.00 %, *R²* = 0.787, and *Q²* = 0.594 (Fig. 2**C**).

The relative concentration of metabolites with PLS-DA-derived VIP scores > 1.0 in colostrum (Fig. 2**D**) and mature milk (Fig. 2**E**) are presented in Table 3. In colostrum, TRT group

**Table 3:** Concentration of potential biomarker metabolites in sows fed a standard gestation diet from day 85–108 of gestation and standard lactation diet from day 109 of gestation until day 21 of lactation (**CON**) or fed the CON diet top dressed with 1 g/sow/day of *E. gracilis*-derived β-glucan (**TRT**) by lactation stage (colostrum, transient milk, and mature milk) (Least square mean ± SEM).

Metabolites^1^	Colostrum		SEM^2^	*P*-value	Transient milk		SEM	*P*-value	Mature milk		SEM	*P*-value
	CON	TRT			CON	TRT			CON	TRT		
Alcohols and polyols												
Methanol	6.904^b^	7.579^a^	0.121	0.001	6.759	6.871	0.125	0.535	6.914	6.615	0.106	0.061
Amines												
Carnitine	8.063^b^	8.403^a^	0.102	0.030	8.000	8.170	0.079	0.125	8.129^a^	7.909^b^	0.070	0.040
Choline	8.098^b^	8.448^a^	0.100	0.024	8.022	8.204	0.080	0.127	8.162^a^	7.939^b^	0.071	0.039
O-Acetylcarnitine	7.624^b^	8.254^a^	0.119	0.002	7.473	7.677	0.124	0.258	7.657	7.448	0.094	0.134
O-phosphocholine	8.098^b^	8.448^a^	0.100	0.024	8.022	8.204	0.080	0.127	8.162^a^	7.939^b^	0.071	0.039
sn-Glycero−3-phosphocoline	8.245^b^	8.759^a^	0.098	0.002	8.175	8.333	0.084	0.202	8.298	8.081	0.077	0.061
Amino acids and derivatives												
Adenine	6.942^b^	7.732^a^	0.177	0.005	6.730	6.995	0.208	0.368	6.937	6.680	0.114	0.127
Alanine	7.448^b^	8.243^a^	0.138	< 0.001	7.252	7.434	0.150	0.405	7.462	7.229	0.116	0.172
Creatine	8.266^b^	8.547^a^	0.050	< 0.001	8.235	8.334	0.054	0.208	8.344	8.319	0.034	0.612
Hypoxanthine	6.694^b^	7.732^a^	0.167	0.005	6.730	6.995	0.208	0.368	6.937	6.680	9.114	0.127
Leucine	8.179^b^	8.976^a^	0.150	0.001	7.987	8.228	0.158	0.295	8.222	7.996	0.115	0.183
N-Acetylglutamate	7.946^b^	8.576^a^	0.123	0.002	7.843	8.058	0.119	0.220	8.000	7.808	0.094	0.166
Threonine	7.161^b^	7.979^a^	0.138	< 0.001	7.000	7.133	0.152	0.545	7.186	6.973	0.113	0.119
Uracil	6.861^b^	7.595^a^	0.173	0.007	6.754	6.894	0.213	0.640	6.847	6.505	0.131	0.081
Carbohydrates and derivatives												
N-Acetylglucosamine	8.042^b^	8.724^a^	0.136	0.002	7.931	8.161	0.130	0.226	8.108	7.874	0.101	0.118
Ribose	6.535^b^	7.104^a^	0.122	0.004	6.337	6.315	0.187	0.935	6.552	6.045	0.135	0.054
UDP-Galactose	7.091^b^	7.788^a^	0.151	0.004	7.120	7.266	0.128	0.422	7.186^a^	6.808^b^	0.101	0.017
UDP-Glucose	7.192^b^	7.865^a^	0.141	0.003	7.189	7.324	0.125	0.455	7.252^a^	6.916^b^	0.100	0.028
UDP-N-Acetylglucosamine	7.858^b^	8.447^a^	0.116	0.002	7.793	7.956	0.108	0.299	7.870	7.621	0.089	0.063
Organic acids												
Acetate	7.488^b^	8.158^a^	0.135	0.003	7.329	7.602	0.140	0.185	7.549	7.377	0.098	0.229
Biotin	7.168^b^	7.947^a^	0.149	0.002	7.013	7.235	0.152	0.314	7.212	6.881	0.117	0.061
Citrate	7.933^b^	8.559^a^	0.110	< 0.001	7.845	7.988	0.103	0.338	7.962	7.764	0.094	0.153
Dimethylamine	7.325^b^	8.034^a^	0.114	< 0.001	7.240	7.410	0.109	0.282	7.357	7.165	0.095	0.169
Lactate	7.351^b^	8.166^a^	0.140	< 0.001	7.184	7.351	0.155	0.458	7.381	7.167	0.113	0.197
cis-Aconitate	7.507^b^	7.861^a^	0.092	0.014	7.428	7.635	0.088	0.115	7.519^a^	7.283^b^	0.074	0.038

^a, b^ Different superscript letters within rows indicate significant differences (*P* < 0.05).

1 Metabolite contents are expressed as log10 [peak area of respective compound in arbitrary unit].

2 Greatest standard error of the mean (SEM).

exhibited higher concentrations of methanol (*P* = 0.001), O-acetylcarnitine (*P* = 0.002), adenine (*P* = 0.005), alanine (*P* < 0.001), hypoxanthine (*P* = 0.005), leucine (*P* = 0.001), N-acetylglutamate (*P* = 0.002), threonine (*P* < 0.001), uracil (*P* = 0.007), N-acetylglucosamine (GlcNAc) (*P* = 0.002), ribose (*P* = 0.004), UDP-galactose (*P* = 0.004), UDP-glucose (*P* = 0.003), UDP-N-acetylglucosamine (UDP-GlcNAc) (*P* = 0.002), acetate (*P* = 0.003), biotin (*P* = 0.002), citrate (*P* < 0.001), dimethylamine (*P* < 0.001), and lactate (*P* < 0.001) than the CON group (Table 3), as potential metabolite biomarkers. In mature milk, the TRT group exhibited lower concentrations of carnitine (*P* = 0.040), choline (*P* = 0.039), O-phosphocholine (*P* = 0.039), UDP-galactose (*P* = 0.017), UDP-glucose (*P* = 0.028), and cis-aconitate (*P* = 0.038) than CON group as potential metabolite biomarkers.

**Fig. 3:**
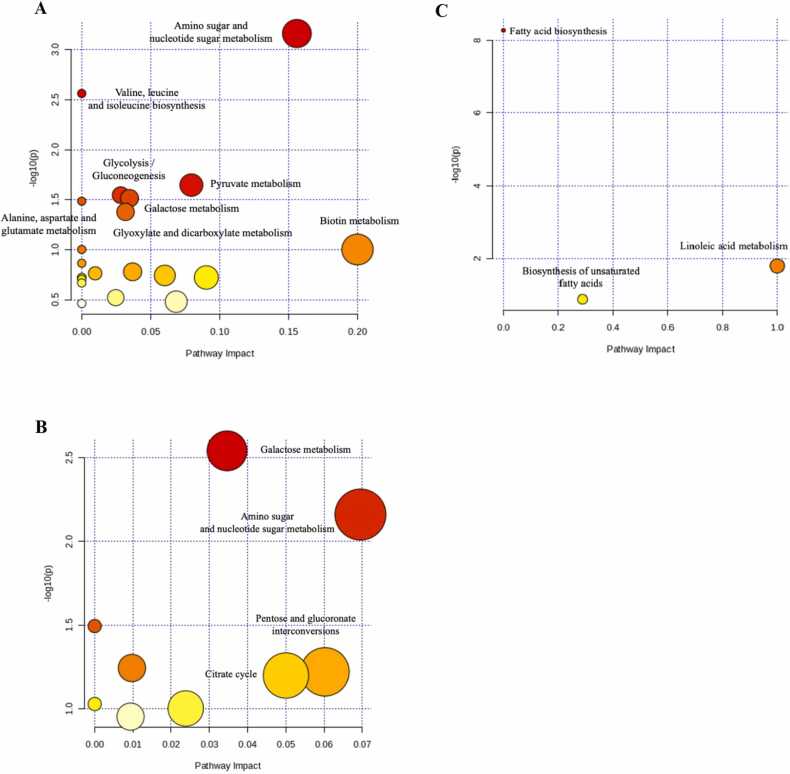
Pathway analysis of metabolite (colostrum; panel **A**, mature milk; panel **B**) and fatty acid (colostrum; panel **C**) synthesis in sows fed a standard gestation diet from day 85–108 of gestation and standard lactation diet from day 109 of gestation until day 21 of lactation (**CON**) or fed the CON diet top dressed with 1 g/sow/day of *E. gracilis*-derived β-glucan (**TRT**). The x-axis indicates the pathway impact and the y-axis indicates the pathway enrichment. Bubble with darker color and larger size represent higher *P*-values from enrichment analysis and greater impact from the pathway topology analysis, respectively.

The *in silico* pathway analysis demonstrated relevant metabolic pathways associated with *E. gracilis* supplementation in colostrum and mature milk. In colostrum, pathways related to amino sugar and nucleotide sugar metabolism, glycolysis/gluconeogenesis, and pyruvate metabolism were modulated (Fig. 3**A**). In mature milk, pathways involving galactose metabolism and amino sugar and nucleotide sugar metabolism were identified (Fig. 3**B**).

### 3.3 Fatty acids profiles of sow colostrum and milk

**Fig. 4:**
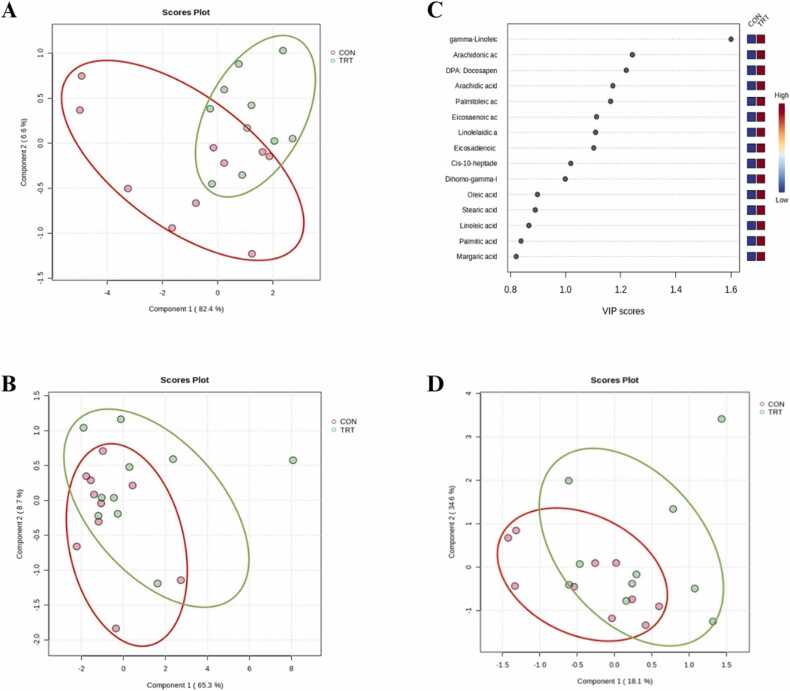
PLS-DA scores plot for the comparison of fatty acid profiles of colostrum (panel **A**), transient milk (panel **B**), and mature milk (panel **D**) samples collected from sows fed a standard gestation diet from day 85–108 of gestation and standard lactation diet from day 109 of gestation until day 21 of lactation (**CON**; red color) or fed the CON diet top dressed with 1 g/sow/day of *E. gracilis*-derived β-glucan (**TRT**; green color). PLS-DA derived VIP scores derived from the comparison among samples of the same day postpartum and indicative potential biomarkers for discrimination in colostrum (panel **C**).

The GC-MS analysis identified 19 FA in colostrum and 23 FA in transient milk and mature milk (Table S2). The PLS-DA scores plots demonstrated clear separation between the groups in colostrum FA profiles, with a prediction accuracy of 80.00 %, *R*² = 0.806, and *Q*² = 0.579 (Fig. 4**A**). In contrast, no distinct patterns were observed in transient milk (Fig. 4**B**) or mature milk (Fig. 4**D**).

Fatty acids with PLS-DA-derived VIP scores > 1.0 in colostrum (Fig. 4**C**) are presented in Table 4. The TRT group showed higher colostrum concentrations of arachidic acid (*P* = 0.028), palmitoleic acid (*P* = 0.033), cis-10-heptadecanoic acid (*P* = 0.035), eicosaenoic acid (*P* = 0.042), docosapentaenoic acid (*P* = 0.027), linolelaidic acid (*P* = 0.022), gamma-linolenic acid (*P* = 0.047), eicosadienoic acid (*P* = 0.025), and arachidonic acid (*P* = 0.019) than CON group (Table 4), as potential FA biomarkers, whereas difference was not detected in transient milk and mature milk. Furthermore, *in silico* pathway analysis indicated modulation of linoleic acid metabolism, FA biosynthesis, and the biosynthesis of unsaturated FA (Fig. 3**C**).

**Table 4:** Concentration of potential biomarker fatty acids in sows fed a standard gestation diet from day 85–108 of gestation and standard lactation diet from day 109 of gestation until day 21 of lactation (**CON**) or fed the CON diet top dressed with 1 g/sow/day of *E. gracilis*-derived β-glucan (**TRT**) by lactation stage (colostrum, transient milk, and mature milk) (Least square mean ± SEM).

Fatty acid^1^	Colostrum		SEM^2^	*P*-value	Transient milk		SEM	*P*-value	Mature milk		SEM	*P*-value
	CON	TRT			CON	TRT			CON	TRT		
Saturated fatty acids (SFA)												
Arachidic acid (C20:0)	6.515^b^	6.688^a^	0.051	0.028	6.807	6.791	0.039	0.780	6.510	6.442	0.059	0.431
Monounsaturated fatty acids (MUFA)												
Palmitoleic acid (C16:1)	7.256^b^	7.430^a^	0.053	0.033	8.033	8.041	0.034	0.870	8.032	7.934	0.051	0.198
Cis−10-heptadecarnoic acid (C17:1)	6.712^b^	6.863^a^	0.047	0.035	6.872	6.854	0.093	0.896	6.459	6.388	0.061	0.422
Eicosaenoic acid (C20:1n9)	6.628^b^	6.794^a^	0.054	0.042	7.065	7.078	0.059	0.875	6.595	6.594	0.078	0.369
Omega−3 polyunsaturated fatty acids (PUFA)												
Docosapentaenoic acid (C22:5n3)	6.494^b^	6.676^a^	0.053	0.027	6.540	6.621	0.043	0.200	6.137	6.001	0.060	0.126
Omega−6 polyunsaturated fatty acids (PUFA)												
Linolelaidic acid (C18:2n6t)	6.441^b^	6.606^a^	0.047	0.022	6.726	6.759	0.054	0.670	6.229	6.289	0.055	0.447
gamma-Linolenic acid (C18:3n6)	6.657^b^	6.878^a^	0.073	0.047	6.957	7.013	0.071	0.584	6.234	6.192	0.080	0.715
Eicosadienoic acid (C20:2n6)	6.834^b^	6.999^a^	0.048	0.025	7.082	7.101	0.058	0.814	6.597	6.534	0.064	0.491
Arachidonic acid (C20:4n6)	7.166^b^	7.351^a^	0.051	0.019	7.245	7.314	0.039	0.235	6.857	6.782	0.055	0.347

^a, b^ Different superscript letters within rows indicate significant differences (*P* < 0.05).

1 Fatty acid contents are expressed as mg/100 g.

2 Greatest standard error of the mean (SEM).

## 4 Discussion

### 4.1 Effect of *E. gracilis* on non-volatile polar metabolite profiles of sow colostrum and milk

Colostrum is essential for neonatal piglet survival and growth, providing critical nutrients and bioactive compounds [2]. Elevated colostrum casein levels have been associated with improved piglet ADG by supplying essential and non-essential AA [3], [4]. de Carvalho et al. [7] reported that supplementation with *E. gracilis* enhances colostrum quantity and quality, as well as piglet growth. In this study, maternal *E. gracilis* supplementation increased the concentrations of multiple NVM and FA in colostrum. Notably, specific metabolites-leucine, threonine, GlcNAc, gamma-linolenic acid, and arachidonic acid-were identified as potential biomarkers and are discussed in relation to their possible direct impact on piglet ADG.

The composition of colostrum and milk metabolites is influenced by multiple factors, including lactation stage, breed, parity, health status, and dietary supplementation [9], [10], [11], [12], [13], [16]. Supplementing late-gestating sows with AA, prebiotics, or probiotics has been shown to positively modulate colostrum and milk metabolite profiles, ultimately leading to enhanced piglet performance [13], [17], [18]. As a prebiotic, *E. gracilis* likely increases the availability of AA for mammary gland uptake from the bloodstream, contributing to improved colostrum and milk metabolite profiles and supporting neonatal development.

Newborn piglets rely on colostrum intake for passive immunity [19], [20], as it provides IgG, which is essential for survival and infection prevention [1], [2], [21]. Colostrum IgG is the predominant immunoglobulin which is largely absorbed through the small intestine via endocytosis by enterocytes within the first 24 h postpartum, providing systemic immunity against pathogens in the early-life until active immunity develops [1], [19], [20], [21]. The majority of colostrum IgG is synthesized before farrowing and transported to the mammary glands via the bloodstream [22]. Threonine, a key component of plasma gamma-globulins, plays a crucial role in colostrum IgG production [23], [24], [25]. This study, along with a previous report [7], confirmed that *E. gracilis* supplementation increases colostrum IgG concentration. This suggests that the effect may be mediated by an enhancement in colostrum threonine levels.

Maternal *E. gracilis* supplementation elevated concentrations of leucine and GlcNAc, both of which are associated with improved piglet growth and gut development. Leucine enhances muscle protein synthesis and gut maturation by increasing villus height and crypt depth [26], [27], which are key factors for effective nutrient absorption [28]. Similarly, GlcNAc, a porcine milk oligosaccharide (OS), supports gut development and absorption [29]. These findings align with previous studies by Heim et al. [30], who demonstrated that algae-derived β-glucan improves gut morphology and increases ADG. Notably, these positive metabolic alterations were primarily observed in colostrum, emphasizing its critical role in early neonatal development. In contrast, mature milk from *E. gracilis*-supplemented sows exhibited reduced concentrations of UDP-Galactose and UDP-Glucose, which are key substrates for piglet growth [31], [32]. Despite these reductions, piglets suckled *E. gracilis*-supplemented sows demonstrated improved ADG. This suggests that the enhanced colostrum metabolites, critical during the neonatal period, outweigh the observed reductions in mature milk metabolites, indicating the importance of *E. gracilis* supplementation during early lactation.

The *In silico* pathway analysis revealed that *E. gracilis* supplementation modulated amino sugar and nucleotide sugar metabolism in colostrum. Amino sugars, such as GlcNAc, are involved in milk OS metabolism and serve as structural components of bacterial cell walls [33], [34]. Similarly, nucleotide sugars like UDP-Galactose, UDP-Glucose, and UDP-GlcNAc play essential roles in gut development and energy utilization [32], [35]. These metabolic modifications suggest enhanced microbial activity in the mammary gland and improved milk OS composition. Furthermore, glucose-related metabolism was modulated by *E. gracilis* supplementation, potentially supporting energy homeostasis during farrowing via pathways such as glycolysis, gluconeogenesis, and pyruvate metabolism, which rely on liver glycogen reserves [19], [36]. Glycolysis converts glucose to pyruvate to produce energy, while gluconeogenesis synthesizes glucose from precursors such as pyruvate, both essential for energy production and regulation during farrowing [37], [38]. These metabolic adaptations reflect dynamic glucose metabolism in *E. gracilis*-supplemented sows to meet the high energy demands of farrowing. For neonatal piglets with limited glycogen reserves [39], external energy sources are important for thermoregulation. The observed modulation in glucose-related metabolism likely supports these energy demands, promoting neonatal vitality. However, further research is necessary to elucidate the metabolic shifts across lactation stages and their underlying mechanisms.

The metabolic modulation observed in colostrum and mature milk was not evident in transient milk, potentially due to its higher fat content. Theil et al. [40] reported that fat content is lowest in colostrum, highest in transient milk, and intermediate in mature milk. This elevated fat content in transient milk reflects a physiological adaptation to meet neonatal piglets' energy demands [40] and is driven by shifts in milk triglycerides and phosphatidylglycerol, which regulate mammary gland metabolism [41]. Since milk metabolite levels negatively correlate with fat content [42], this may explain the minimal metabolic changes in transient milk. Similarly, Ruampatana et al. [13] observed minimal metabolite alterations in transient milk after probiotic supplementation, further suggesting that its high fat content may limit metabolic modulation.

### 4.2 Effect of *E. gracilis* on fatty acid profiles of sow colostrum and milk

Milk fat, one of the primary nutrient sources for newborn piglets [43], is secreted in the form of milk fat globules (MFG), which primarily composed of triglycerides with small amounts of free FA [44], [45]. The composition and properties of milk fat are influenced by dietary fat sources and MFG diameter [46], [47]. Additionally, supplementation with prebiotics and probiotics in late-gestating sows has been shown to modulate FA profiles [13], [18], [48]. In this study, *E. gracilis* supplementation increased colostrum FA levels, such as gamma-linolenic acid and arachidonic acid, likely reflecting the modulatory effect of *E. gracilis* supplementation on the profile of FA. Similarly, maternal supplementation of laminarin, another β-glucan derived from algae, has been shown to enhance specific milk FA levels, such as capric acid, myristic acid, and palmitoleic acid [18], highlighting the role of β-glucan sources in shaping milk FA profiles. Gamma-linolenic acid may support piglet health through its anti-inflammatory and immune-supporting properties [49]. Arachidonic acid has been associated with enhanced immunity and growth in neonatal piglets and growing pigs [50], [51]. These findings suggested that *E. gracilis* supplementation enhances the FA composition of colostrum, potentially benefiting piglet immunity and development.

Linoleic acid, the predominant polyunsaturated FA in pig muscle [52], [53], undergoes metabolism that involves gut-derived conjugated linoleic acid (CLA) [54]. Maternal CLA supplementation has been linked to enhanced immune development in piglets, increasing colostrum IgG levels [55], [56]. Similarly, *E. gracilis* supplementation modulated linoleic acid metabolism, potentially promoting endogenous CLA production and facilitating IgG transfer from bloodstream to mammary gland. These findings indicate the role of *E. gracilis* in optimizing colostrum Ig levels; however, further studies are needed to elucidate the precise mechanisms regulating CLA metabolism and its impact on colostrum immunoglobulin G concentration.

## 5 Conclusions

Maternal supplementation with *E. gracilis*-derived β-glucan during late gestation and lactation sows positively impacts colostrum metabolite and fatty acid profiles, potentially enhancing piglet ADG. Metabolomic and lipidomic analyses identified key biomarkers in colostrum, including leucine, GlcNAc, threonine, gamma-linolenic acid, and arachidonic acid, indicating improved colostrum quality. These findings support the hypothesis that *E. gracilis* optimizes mammary gland secretions, contributing to piglet growth. While *E. gracilis* appears to be a promising prebiotic for improving colostrum biomolecular composition and piglet performance, further studies are needed to validate its long-term effects on immune function and metabolic development.

## CRediT authorship contribution statement

**Jakavat Ruampatana:** Conceptualization, Data curation, Formal analysis, Investigation, Methodology, Validation, Writing – original draft, Writing – review & editing. **Takele Feyera:** Data curation, Methodology, Validation, Writing – review & editing. **Unchean Yamsrikaew:** Data curation, Investigation. **Methaporn Juarjan:** Formal analysis, Validation. **Kunaporn Homyog:** Formal analysis, Validation. **Wanwimon Mekboonsonglarp:** Formal analysis, Validation. **Sarn Settachaimongkon:** Data curation, Formal analysis, Methodology, Validation, Writing – review & editing. **Morakot Nuntapaitoon:** Conceptualization, Data curation, Investigation, Methodology, Project administration, Supervision, Writing – review & editing.

## Declaration of Competing Interest

The authors declare that they have no known competing financial interests or personal relationships that could have appeared to influence the work reported in this paper.

## Data Availability

The datasets used and/or analyzed during the current study are available from the corresponding author upon reasonable request.
