# Supplementary material for: Metabolomic and lipidomic insights into the impact of Euglena gracilis-derived β-glucan supplementation on sow colostrum and milk composition
Source: Comput Struct Biotechnol J. 2025 Feb 27;27:869–78. doi: 10.1016/j.csbj.2025.02.033 (PMC11925092; doi:10.1016/j.csbj.2025.02.033)
Supplement: Supplementary file 1 — Supplementary material [file mmc1.docx]

**Supplementary data**

**Table S1** Concentration of non-volatile polar metabolites in sows fed a standard gestation diet from day 85 to 108 of gestation and standard lactation diet from day 109 of gestation until day 21 of lactation (**CON**) or fed the CON diet top dressed with 1 g/sow/day of *E. gracilis*-derived β-glucan (**TRT**) by lactation stage (colostrum, transient milk, and mature milk) (Least square mean ± SEM).

| Metabolites^1^ | Colostrum | | SEM | *P*-value | Transient milk | | | SEM | *P*-value | | Mature milk | | | SEM | *P*-value |
| --- | --- | --- | --- | --- | --- | --- | --- | --- | --- | --- | --- | --- | --- | --- | --- |
|  | CON | TRT |  |  | CON | TRT | |  |  |  | CON | | TRT |  |  |
| Alcohols and polyols | | | | | | | | | | | | | | | |
| Arabitol | 8.626^b^ | 8.794^a^ | 0.055 | 0.045 | 8.612 | 8.705 | | 0.062 | 0.306 | | 8.728 | | 8.671 | 0.030 | 0.193 |
| Erythritol | 8.815 | 8.944 | 0.056 | 0.119 | 8.799 | 8.890 | | 0.063 | 0.318 | | 8.921 | | 8.864 | 0.029 | 0.181 |
| Ethanol | 9.093 | 9.225 | 0.057 | 0.092 | 9.066 | 9.165 | | 0.063 | 0.280 | | 9.187 | | 9.131 | 0.030 | 0.196 |
| Methanol | 6.904^b^ | 7.579^a^ | 0.121 | 0.001 | 6.759 | 6.871 | | 0.125 | 0.535 | | 6.914 | | 6.615 | 0.106 | 0.061 |
| Myo-Inositol | 8.488^b^ | 8.785^a^ | 0.062 | 0.003 | 8.471 | 8.551 | | 0.062 | 0.376 | | 8.578 | | 8.492 | 0.039 | 0.134 |
| Amines | | | | | | | | | | | | | | | |
| Carnitine | 8.063^b^ | 8.403^a^ | 0.102 | 0.030 | 8.000 | 8.170 | | 0.079 | 0.125 | | 8.129^a^ | | 7.909^b^ | 0.070 | 0.040 |
| Choline | 8.098^b^ | 8.448^a^ | 0.100 | 0.024 | 8.022 | 8.204 | | 0.080 | 0.127 | | 8.162^a^ | | 7.939^b^ | 0.071 | 0.039 |
| O-Acetylcarnitine | 7.624^b^ | 8.254^a^ | 0.119 | 0.002 | 7.473 | 7.677 | | 0.124 | 0.258 | | 7.657 | | 7.448 | 0.094 | 0.134 |
| O-Acteylcholine | 8.209^b^ | 8.669^a^ | 0.101 | 0.005 | 8.123 | 8.295 | | 0.089 | 0.188 | | 8.262 | | 8.050 | 0.077 | 0.066 |
| O-phosphocholine | 8.098^b^ | 8.448^a^ | 0.100 | 0.024 | 8.022 | 8.204 | | 0.080 | 0.127 | | 8.162^a^ | | 7.939^b^ | 0.071 | 0.039 |
| sn-Glycero-3-phosphocoline | 8.245^b^ | 8.759^a^ | 0.098 | 0.002 | 8.175 | 8.333 | | 0.084 | 0.202 | | 8.298 | | 8.081 | 0.077 | 0.061 |
| Amino acids and derivatives | | | | | | | | | | | | | | | |
| Adenine | 6.942^b^ | 7.732^a^ | 0.177 | 0.005 | 6.730 | 6.995 | | 0.208 | 0.368 | | 6.937 | | 6.680 | 0.114 | 0.127 |
| Alanine | 7.448^b^ | 8.243^a^ | 0.138 | <0.001 | 7.252 | 7.434 | | 0.150 | 0.405 | | 7.462 | | 7.229 | 0.116 | 0.172 |
| Betain | 8.555^b^ | 8.844^a^ | 0.07 | 0.010 | 8.522 | 8.632 | | 0.065 | 0.250 | | 8.644 | | 8.519 | 0.046 | 0.071 |
| Creatine | 8.266^b^ | 8.547^a^ | 0.050 | <0.001 | 8.235 | 8.334 | | 0.054 | 0.208 | | 8.344 | | 8.319 | 0.034 | 0.612 |
| Creatinine | 7.799^b^ | 8.333^a^ | 0.101 | 0.002 | 7.739 | 7.829 | | 0.092 | 0.504 | | 7.828 | | 7.607 | 0.082 | 0.074 |
| Creatine phosphate | 8.706 | 8.814 | 0.051 | 0.154 | 8.687 | 8.793 | | 0.061 | 0.234 | | 8.809 | | 8.778 | 0.026 | 0.403 |
| Glutamate | 8.784 | 8.921 | 0.052 | 0.080 | 8.769 | 8.870 | | 0.062 | 0.259 | | 8.889 | | 8.844 | 0.028 | 0.263 |
| Glycine | 8.527 | 8.600 | 0.052 | 0.345 | 8.517 | 8.624 | | 0.061 | 0.233 | | 8.635 | | 8.605 | 0.025 | 0.497 |
| Hypoxanthine | 6.694^b^ | 7.732^a^ | 0.167 | 0.005 | 6.730 | 6.995 | | 0.208 | 0.368 | | 6.937 | | 6.680 | 9.114 | 0.127 |
| Inosine | 8.800^b^ | 9.015^a^ | 0.060 | 0.021 | 8.784 | 8.875 | | 0.063 | 0.325 | | 8.897 | | 8.819 | 0.035 | 0.136 |
| Isoleucine | 8.868^b^ | 9.041^a^ | 0.056 | 0.044 | 8.850 | 8.943 | | 0.063 | 0.315 | | 8.970 | | 8.912 | 0.030 | 0.197 |
| Leucine | 8.179^b^ | 8.976^a^ | 0.150 | 0.001 | 7.987 | 8.228 | | 0.158 | 0.295 | | 8.222 | | 7.996 | 0.115 | 0.183 |
| N-Acetylglutamate | 7.946^b^ | 8.576^a^ | 0.123 | 0.002 | 7.843 | 8.058 | | 0.119 | 0.220 | | 8.000 | | 7.808 | 0.094 | 0.166 |
| Threonine | 7.161^b^ | 7.979^a^ | 0.138 | <0.001 | 7.000 | 7.133 | | 0.152 | 0.545 | | 7.186 | | 6.973 | 0.113 | 0.119 |
| Taurine | 7.267^b^ | 7.764^a^ | 0.087 | <0.001 | 7.159 | 7.162 | | 0.093 | 0.981 | | 7.282 | | 7.092 | 0.074 | 0.085 |
| UMP | 7.848^b^ | 8.354^a^ | 0.101 | 0.002 | 7.785 | 7.991 | | 0.092 | 0.130 | | 7.857 | | 7.685 | 0.082 | 0.157 |
| Uracil | 6.861^b^ | 7.595^a^ | 0.173 | 0.007 | 6.754 | 6.894 | | 0.213 | 0.640 | | 6.847 | | 6.505 | 0.131 | 0.081 |
| Uridine | 8.497^b^ | 8.760^a^ | 0.066 | 0.013 | 8.483 | 8.564 | | 0.065 | 0.385 | | 8.590 | | 8.486 | 0.041 | 0.091 |
| Valine | 8.979^b^ | 9.312^a^ | 0.068 | 0.003 | 8.942 | 9.021 | | 0.068 | 0.426 | | 9.064 | | 8.963 | 0.046 | 0.135 |
| 4-Carboxyglutamate | 8.824^b^ | 9.035^a^ | 0.061 | 0.025 | 8.810 | 8.886 | | 0.064 | 0.411 | | 8.922 | | 8.842 | 0.035 | 0.123 |
| Carbohydrates and derivatives | | | | | | | | | | | | | | | |
| Lactose | 9.700 | 9.847 | 0.055 | 0.074 | 9.684 | | 9.778 | 0.062 | | 0.295 | | 9.800 | 9.747 | 0.029 | 0.216 |
| N-Acetylglucosamine | 8.042^b^ | 8.724^a^ | 0.136 | 0.002 | 7.931 | | 8.161 | 0.130 | | 0.226 | | 8.108 | 7.874 | 0.101 | 0.118 |
| Ribose | 6.535^b^ | 7.104^a^ | 0.122 | 0.004 | 6.337 | | 6.315 | 0.187 | | 0.935 | | 6.552 | 6.045 | 0.135 | 0.054 |
| UDP-Galactose | 7.091^b^ | 7.788^a^ | 0.151 | 0.004 | 7.120 | | 7.266 | 0.128 | | 0.422 | | 7.186^a^ | 6.808^b^ | 0.101 | 0.017 |
| UDP-Glucose | 7.192^b^ | 7.865^a^ | 0.141 | 0.003 | 7.189 | | 7.324 | 0.125 | | 0.455 | | 7.252^a^ | 6.916^b^ | 0.100 | 0.028 |
| UDP-N-Acetylglucosamine | 7.858^b^ | 8.447^a^ | 0.116 | 0.002 | 7.793 | | 7.956 | 0.108 | | 0.299 | | 7.870 | 7.621 | 0.089 | 0.063 |
| Organic acids | | | | | | | | | | | | | | | |
| Acetate | 7.488^b^ | 8.158^a^ | 0.135 | 0.003 | 7.329 | | 7.602 | 0.140 | | 0.185 | | 7.549 | 7.377 | 0.098 | 0.229 |
| Biotin | 7.168^b^ | 7.947^a^ | 0.149 | 0.002 | 7.013 | | 7.235 | 0.152 | | 0.314 | | 7.212 | 6.881 | 0.117 | 0.061 |
| Citrate | 7.933^b^ | 8.559^a^ | 0.110 | <0.001 | 7.845 | | 7.988 | 0.103 | | 0.338 | | 7.962 | 7.764 | 0.094 | 0.153 |
| Dimethylamine | 7.325^b^ | 8.034^a^ | 0.114 | <0.001 | 7.240 | | 7.410 | 0.109 | | 0.282 | | 7.357 | 7.165 | 0.095 | 0.169 |
| Glycolate | 8.732 | 8.852 | 0.052 | 0.119 | 8.716 | | 8.821 | 0.061 | | 0.238 | | 8.837 | 8.802 | 0.026 | 0.351 |
| Lactate | 7.351^b^ | 8.166^a^ | 0.140 | <0.001 | 7.184 | | 7.351 | 0.155 | | 0.458 | | 7.381 | 7.167 | 0.113 | 0.197 |
| 2-Hydroxyisovalerate | 8.605^b^ | 8.993^a^ | 0.079 | 0.003 | 8.555 | | 8.675 | 0.076 | | 0.280 | | 8.682 | 8.561 | 0.054 | 0.131 |
| cis-Aconitate | 7.507^b^ | 7.861^a^ | 0.092 | 0.014 | 7.428 | | 7.635 | 0.088 | | 0.115 | | 7.519^a^ | 7.283^b^ | 0.074 | 0.038 |

^1^ Metabolite contents are expressed as log10 [peak area of respective compound in arbitrary unit].

^2^ Greatest standard error of the mean (SEM).

^a, b^ Different superscript letters within rows indicate significant differences (*P* < 0.05).

**Table S2** Concentration of fatty acids in sows fed a standard gestation diet from day 85 to 108 of gestation and standard lactation diet from day 109 of gestation until day 21 of lactation (**CON**) or fed the CON diet top dressed with 1 g/sow/day of *E. gracilis*-derived β-glucan (**TRT**) by lactation stage (colostrum, transient milk, and mature milk) (Least square mean ± SEM).

| Fatty acid^1^ | Colostrum | | SEM | *P*-value | | Transient milk | | SEM | *P*-value | Mature milk | | | SEM | *P*-value |
| --- | --- | --- | --- | --- | --- | --- | --- | --- | --- | --- | --- | --- | --- | --- |
|  | CON | TRT |  |  |  | CON | TRT |  |  | CON | TRT | |  |  |
| Saturated fatty acids (SFA) | | | | | | | | | | | | | | |
| Hexanoic acid (C6:0) |  |  |  |  | | 5.230 | 5.152 | 0.030 | 0.083 | 5.348 | 5.280 | | 0.041 | 0.254 |
| Caprylic acid (C8:0) |  |  |  |  | | 5.341 | 5.315 | 0.045 | 0.696 | 5.601 | 5.484 | | 0.053 | 0.133 |
| Capric acid (C10:0) |  |  |  |  | | 6.036 | 6.090 | 0.059 | 0.530 | 6.441 | 6.324 | | 0.051 | 0.119 |
| Lauric acid (C12:0) | 5.620 | 5.563 | 0.060 | 0.507 | | 6.450 | 6.393 | 0.092 | 0.665 | 6.570 | 6.463 | | 0.054 | 0.177 |
| Myristic acid (C14:0) | 7.183 | 7.263 | 0.047 | 0.246 | | 7.664 | 7.698 | 0.029 | 0.422 | 7.686 | 7.575 | | 0.053 | 0.159 |
| Pentadecanoic acid (C15:0) | 6.334 | 6.42 | 0.041 | 0.160 | | 6.645 | 6.693 | 0.034 | 0.331 | 6.620 | 6.497 | | 0.051 | 0.104 |
| Palmitic acid (C16:0) | 8.488^b^ | 8.613^a^ | 0.040 | 0.040 | | 8.741 | 8.769 | 0.022 | 0.378 | 8.647 | 8.558 | | 0.043 | 0.164 |
| Margaric acid (C17:0) | 6.574 | 6.696 | 0.046 | 0.074 | | 6.830 | 6.917 | 0.036 | 0.108 | 6.469 | 6.409 | | 0.050 | 0.411 |
| Stearic acid (C18:0) | 7.902 | 8.034 | 0.048 | 0.065 | | 8.169 | 8.096 | 0.097 | 0.599 | 7.837 | 7.745 | | 0.053 | 0.239 |
| Arachidic acid (C20:0) | 6.515^b^ | 6.688^a^ | 0.051 | 0.028 | | 6.807 | 6.791 | 0.039 | 0.780 | 6.510 | 6.442 | | 0.059 | 0.431 |
| Behenic acid (C22:0) |  |  |  |  | | 6.094 | 6.113 | 0.039 | 0.732 | 5.884 | 5.891 | | 0.059 | 0.929 |
| Monounsaturated fatty acids (MUFA) | | | | | | | | | | | | | | |
| Palmitoleic acid (C16:1) | 7.256^b^ | 7.430^a^ | 0.053 | 0.033 | | 8.033 | 8.041 | 0.034 | 0.870 | 8.032 | | 7.934 | 0.051 | 0.198 |
| Cis-10-heptadecarnoic acid (C17:1) | 6.712^b^ | 6.863^a^ | 0.047 | 0.035 | | 6.872 | 6.854 | 0.093 | 0.896 | 6.459 | | 6.388 | 0.061 | 0.422 |
| Oleic acid (C18:1n9c) | 8.716^b^ | 8.850^a^ | 0.038 | 0.023 | | 8.986 | 9.008 | 0.036 | 0.667 | 8.676 | | 8.604 | 0.055 | 0.371 |
| Eicosaenoic acid (C20:1n9) | 6.628^b^ | 6.794^a^ | 0.054 | 0.042 | | 7.065 | 7.078 | 0.059 | 0.875 | 6.595 | | 6.594 | 0.078 | 0.369 |
| Omega-3 polyunsaturated fatty acids (PUFA) | | | | | | | | | | | | | | |
| Linolenic acid (C18:3n3) | 7.236 | 7.339 | 0.051 | | 0.172 | 7.468 | 7.473 | 0.045 | 0.936 | 7.413 | | 7.333 | 0.061 | 0.361 |
| Docosapentaenoic acid (C22:5n3) | 6.494^b^ | 6.676^a^ | 0.053 | | 0.027 | 6.540 | 6.621 | 0.043 | 0.200 | 6.137 | | 6.001 | 0.060 | 0.126 |
| Omega-6 polyunsaturated fatty acids (PUFA) | | | | | | | | | | | | | | |
| Linolelaidic acid (C18:2n6t) | 6.441^b^ | 6.606^a^ | 0.047 | | 0.022 | 6.726 | 6.759 | 0.054 | 0.670 | 6.229 | | 6.289 | 0.055 | 0.447 |
| Linoleic acid (C18:2n6c) | 8.543 | 8.672 | 0.045 | | 0.058 | 8.695 | 8.707 | 0.034 | 0.819 | 8.509 | | 8.431 | 0.053 | 0.318 |
| gamma-Linolenic acid (C18:3n6) | 6.657^b^ | 6.878^a^ | 0.073 | | 0.047 | 6.957 | 7.013 | 0.071 | 0.584 | 6.234 | | 6.192 | 0.080 | 0.715 |
| Eicosadienoic acid (C20:2n6) | 6.834^b^ | 6.999^a^ | 0.048 | | 0.025 | 7.082 | 7.101 | 0.058 | 0.814 | 6.597 | | 6.534 | 0.064 | 0.491 |
| Dihomo-gamma-linolenic (C20:3n6) | 6.573 | 6.721 | 0.052 | | 0.059 | 6.689 | 6.698 | 0.052 | 0.910 | 6.132 | | 6.059 | 0.066 | 0.439 |
| Arachidonic acid (C20:4n6) | 7.166^b^ | 7.351^a^ | 0.051 | | 0.019 | 7.245 | 7.314 | 0.039 | 0.235 | 6.857 | | 6.782 | 0.055 | 0.347 |

^1^ Fatty acid contents are expressed as mg/100g.

^2^ Greatest standard error of the mean (SEM).

^a, b^ Different superscript letters within rows indicate significant differences (*P* < 0.05).

**Table S3**. Assignment table of non-volatile polar metabolites present in the ^1^H-NMR spectra of sow mammary gland secretion (colostrum, transient milk, and mature milk)

| Chemical group | Number | Compound name | ^1^H chemical shift, ppm^a^ |
| --- | --- | --- | --- |
| Alcohols and polyols | 1 | Arabitol | 3.73 (m) |
|  | 2 | Erythritol | 3.77 (m) |
|  | 3 | Ethanol | 3.65 (m) |
|  | 4 | Methanol | 3.35 (s) |
|  | 5 | Myo-Inositol | 3.29 (t), 4.07 (t) |
| Amines | 6 | Carnitine | 3.21 (s) |
|  | 7 | Choline | 3.19 (s) |
|  | 8 | O-Acetylcarnitine | 2.13 (s), 3.17 (s) |
|  | 9 | O-Acteylcholine | 2.13 (s), 3.17 (s) |
|  | 10 | O-phosphocholine | 3.21 (s), 3.63 (m), 4.19 (s) |
|  | 11 | sn-Glycero-3-phosphocoline | 3.21 (s), 4.31 (m) |
| Amino acids and derivatives | 12 | Adenine | 8.17 (s), 8.21 (s) |
|  | 13 | Alanine | 1.47 (d) |
|  | 14 | Betain | 3.25 (s), 3.87 (s) |
|  | 15 | Creatine | 3.03 (s), 3.91 (s) |
|  | 16 | Creatinine | 3.03 (s), 4.05 (s) |
|  | 17 | Creatine phosphate | 3.03 (s), 3.93 (s) |
|  | 18 | Glutamate | 3.73 (m) |
|  | 19 | Glycine | 3.55 (s) |
|  | 20 | Hypoxanthine | 8.21 (d) |
|  | 21 | Inosine | 3.83 (m), 6.09 (d), 8.23 (s) |
|  | 22 | Isoleucine | 0.99 (t), 3.67 (d) |
|  | 23 | Leucine | 0.91 (m), 1.71 (m) |
|  | 24 | N-Acetylglutamate | 2.03 (s), 4.11 (m) |
|  | 25 | Threonine | 1.31 (d), 3.59 (d) |
|  | 26 | Taurine | 3.43 (t) |
|  | 27 | UMP | 4.35 (t), 5.97 (m), 8.01 (d) |
|  | 28 | Uracil | 5.79 (d), 7.51 (d) |
|  | 29 | Uridine | 3.77 (m), 3.85 (m), 4.13 (m), 5.91 (t), 7.87 (d), |
|  | 30 | Valine | 3.61 (d) |
|  | 31 | 4-Carboxyglutamate | 3.73 (m) |
| Carbohydrates and derivatives | 32 | Lactose | 3.25 (t), 3.55 (m), 3.67 (m), 3.73 (m), 3.82 (m), 3.95 (m), 4.47 (d), 4.65 (d), 5.23 (d) |
|  | 33 | N-Acetylglucosamine | 2.05 (s), 3.45 (m), 3.53 (m), 8.09 (d) |
|  | 34 | Ribose | 4.09 (s), 5.39 (d) |
|  | 35 | UDP-Galactose | 5.63 (m), 5.93 (t), 7.93 (d) |
|  | 36 | UDP-Glucose | 3.43 (t), 3.53 (m), 4.17 (m),  5.59 (m), 7.91 (d) |
|  | 37 | UDP-N-Acetylglucosamine | 2.07 (s), 4.21 (m), 5.51 (m), 5.95 (t), 7.93 (d), 8.31 (d) |
| Organic acids | 38 | Acetate | 1.91 (s) |
|  | 39 | Biotin | 2.75 (d) |
|  | 40 | Citrate | 2.51 (d), 2.71 (d) |
|  | 41 | Dimethylamine | 2.71 (s) |
|  | 42 | Glycolate | 3.93 (s) |
|  | 43 | Lactate | 1.31 (d) |
|  | 44 | 2-Hydroxyisovalerate | 0.89 (s), 1.73 (s) |
|  | 45 | cis-Aconitate | 3.17 (s), 5.97 (s) |

^a^ Chemical shift values are referenced to TSP signal (0.00 ppm) at pH 6.00; ^b^ Letters indicate singlet (s), doublet (d), triplet (t), and multiplet (m) multiplicity of ^1^H-NMR peak, respectively.

**Table S4**. Retention time of fatty acids present in the GC-MS-FAME of sow mammary gland secretion (colostrum, transient milk, and mature milk).

| Chemical group | Number | Retention time (min) | Compound name | Chemical formula |
| --- | --- | --- | --- | --- |
| Saturated fatty acids (SFA) | 1 | 11.35 | Hexanoic acid | (C6:0) |
|  | 2 | 12.76 | Caprylic acid | (C8:0) |
|  | 3 | 14.23 | Capric acid | (C10:0) |
|  | 4 | 15.63 | Lauric acid | (C12:0) |
|  | 5 | 17.08 | Myristic acid | (C14:0) |
|  | 6 | 17.86 | Pentadecanoic acid | (C15:0) |
|  | 7 | 18.77 | Palmitic acid | (C16:0) |
|  | 8 | 19.70 | Margaric acid | (C17:0) |
|  | 9 | 20.75 | Stearic acid | (C18:0) |
|  | 10 | 23.04 | Arachidic acid | (C20:0) |
|  | 11 | 25.49 | Behenic acid | (C22:0) |
| Monounsaturated fatty acids (MUFA) | 12 | 19.57 | Palmitoleic acid | (C16:1) |
|  | 13 | 20.54 | Cis-10-heptadecarnoic acid | (C17:1) |
|  | 14 | 21.63 | Oleic acid | (C18:1n9c) |
|  | 15 | 23.96 | Eicosaenoic acid | (C20:1n9) |
| Omega-3 polyunsaturated fatty acids (PUFA) | 16 | 24.32 | Linolenic acid | (C18:3n3) |
|  | 17 | 31.62 | Docosapentaenoic acid | (C22:5n3) |
| Omega-6 polyunsaturated fatty acids (PUFA) | 18 | 21.87 | Linolelaidic acid | (C18:2n6t) |
|  | 19 | 22.88 | Linoleic acid | (C18:2n6c) |
|  | 20 | 23.83 | gamma-Linolenic acid | (C18:3n6) |
|  | 21 | 25.28 | Eicosadienoic acid | (C20:2n6) |
|  | 22 | 26.27 | Dihomo-gamma-linolenic | (C20:3n6) |
|  | 23 | 27.05 | Arachidonic acid | (C20:4n6) |


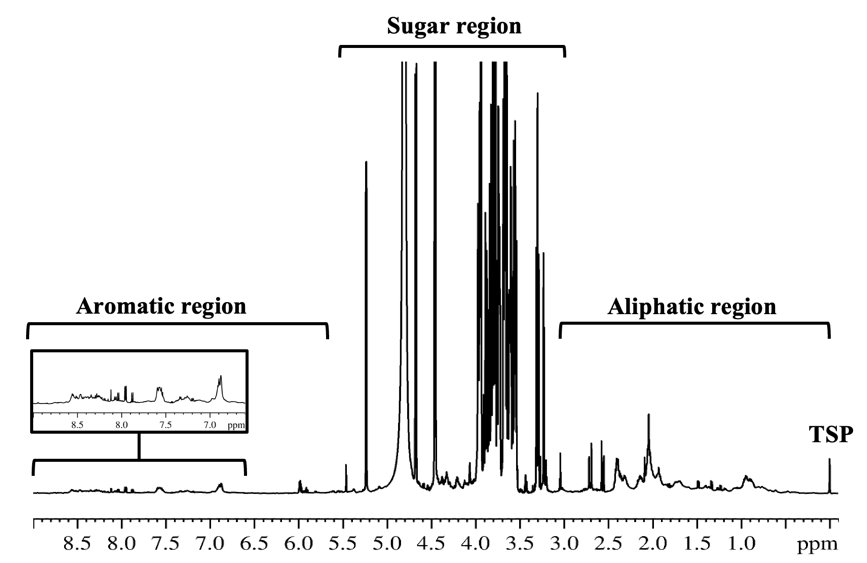


**A**

**B**


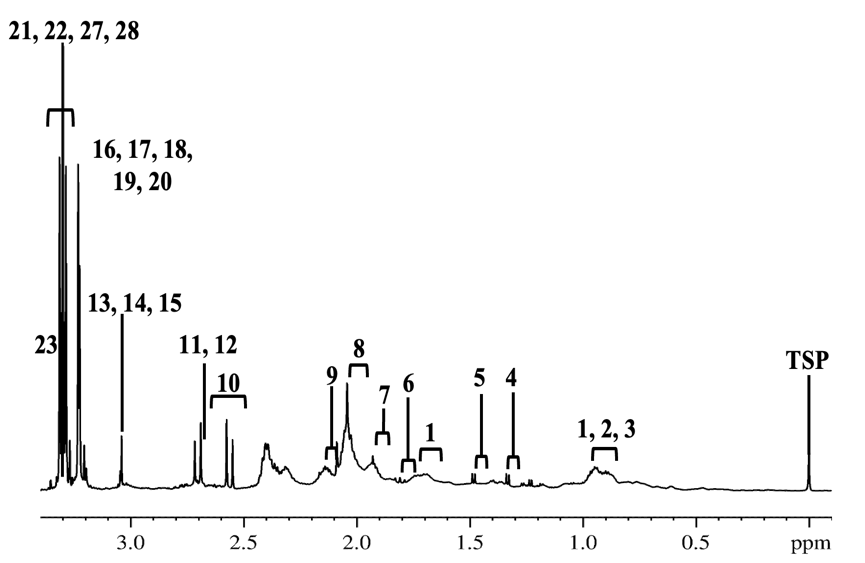


**C**


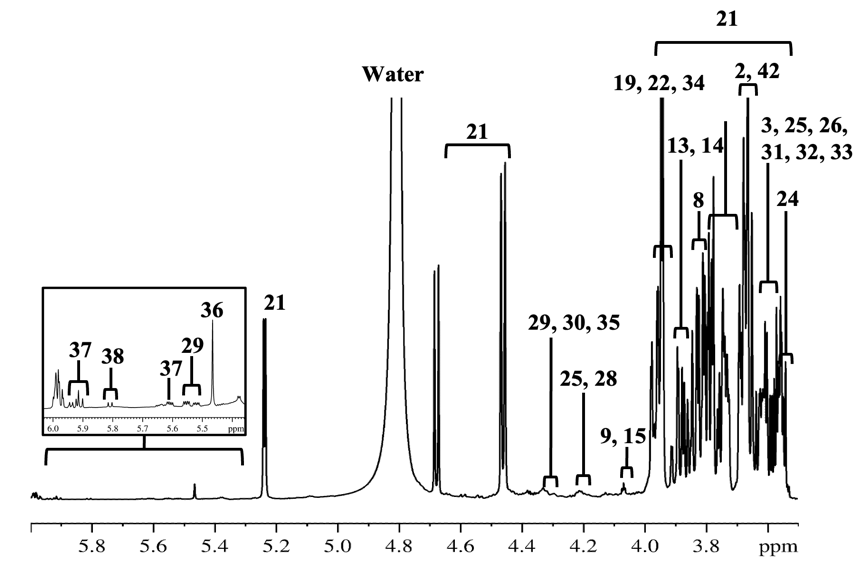


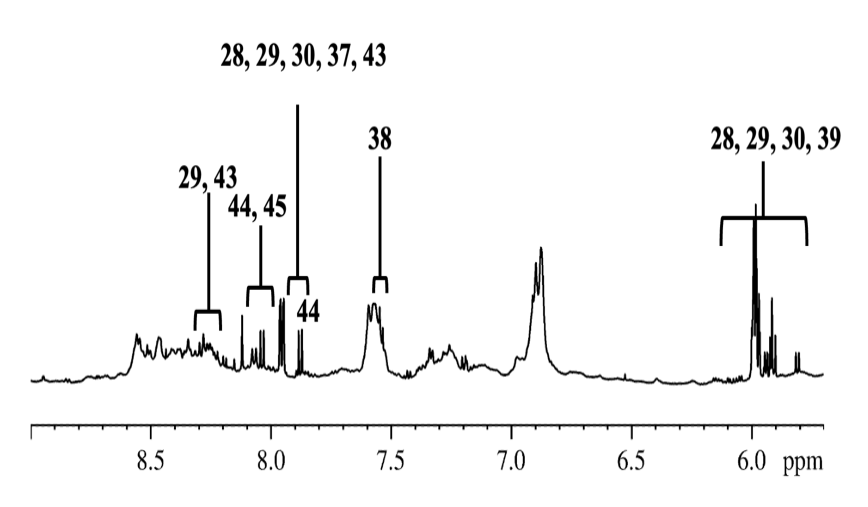


**D**

**Figure S1**. ^1^H-NMR spectra of samples (colostrum, transient milk, mature milk) from sows fed with *E. gracilis*-derived β-glucan supplementation (**A**) and expansions corresponding for aliphatic region (**B**), sugar region (**C**) and aromatic region (**D**) with assigned peaks: TSP: internal standard, 1: leucine, 2: isoleucine, 3: valine, 4: lactate, 5: alanine, 6: 2-hydroxybutyrate, 7: acetate, 8: 2-hydroxyisovalerate, 9: N-acetylglutamate, 10: citrate, 11: dimethylamine, 12: biotin, 13: creatine, 14: creatine phosphate, 15: creatinine, 16: O-acetylcarnitine, 17: O-acetylcholine, 18: betaine, 19: carnitine, 20: choline, 21: lactose, 22: myo-inositol, 23: methanol, 24: glycine, 25: O-phosphocholine, 26: threonine, 27: taurine, 28: UDP-glucose, 29: UDP-N-acetylglucosamine, 30: UMP, 31: arabitol, 32: erythritol, 33: 4-carboxyglutamate, 34: glycolate, 35: sn-glycerol-3-phosphocholine, 36: ribose, 37: UDP-galactose, 38: uracil, 39: cis-aconitate, 40: ethanol, 41: inosine, 42: uridine, 43: N-acetylglucosamine, 44: adenine, 45: hypoxanthine.

**
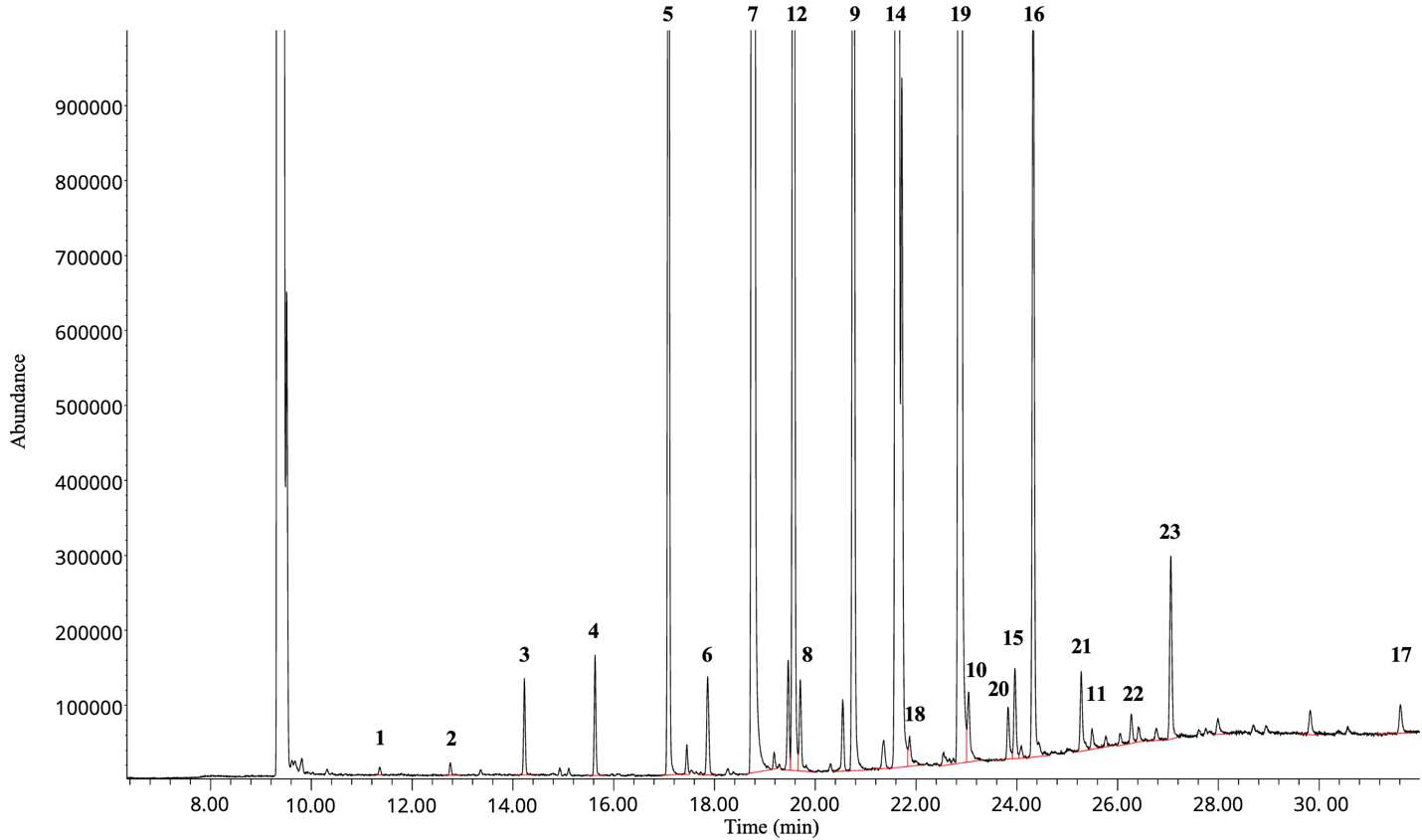
**

**Figure S2**. GC-MS chromatogram of FAMEs of samples (colostrum, transient milk, mature milk) from sows fed with *E. gracilis*-derived β-glucan supplementation. Peak identification: 1: hexanoic acid, 2: caprylic acid, 3: capric acid, 4: lauric acid, 5: myristic acid, 6: pentadecanoic acid, 7: palmitic acid, 8: margaric acid, 9: stearic acid, 10: arachidic acid, 11: behenic acid, 12: palmitoleic acid, 13: cis-10-heptadecarnoic acid, 14: oleic acid, 15: eicosaenoic acid, 16: linolenic acid, 17: docosapentaenoic acid, 18: linolelaidic acid, 19: linoleic acid, 20: gamma-Linolenic acid, 21: eicosadienoic acid, 22: dihomo-gamma-linolenic, 23: arachidonic acid.


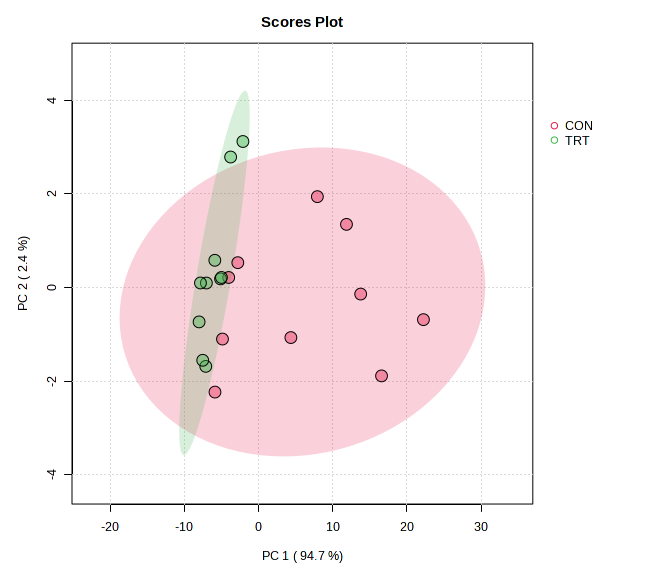

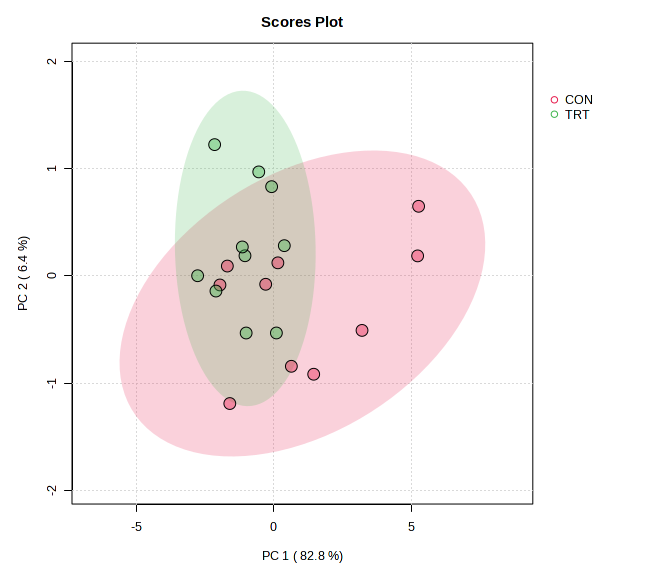


**D**

**A**


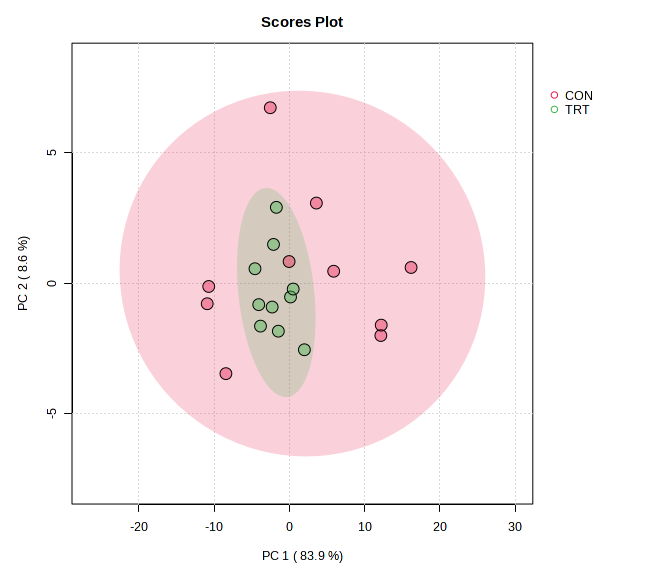

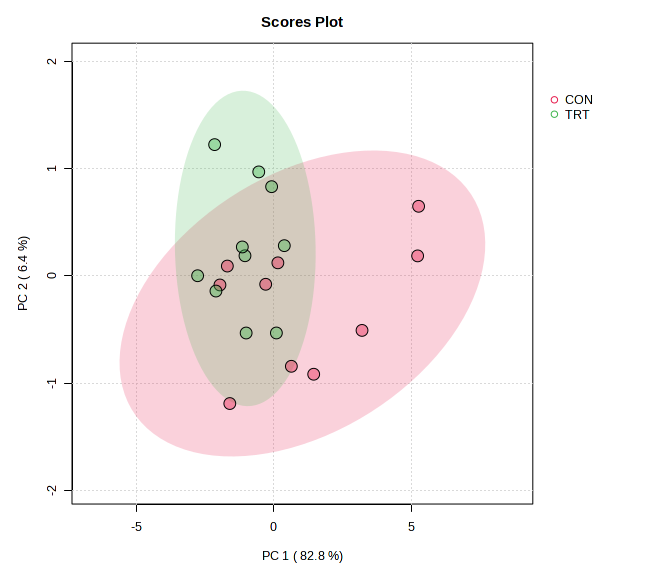


**E**

**B**


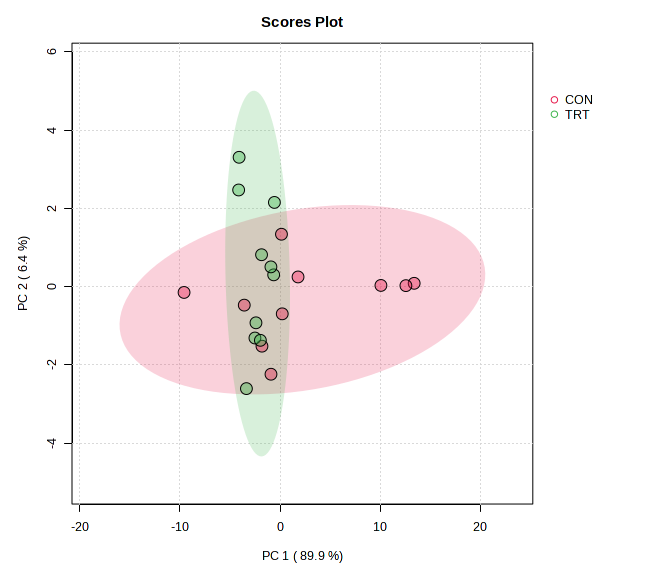

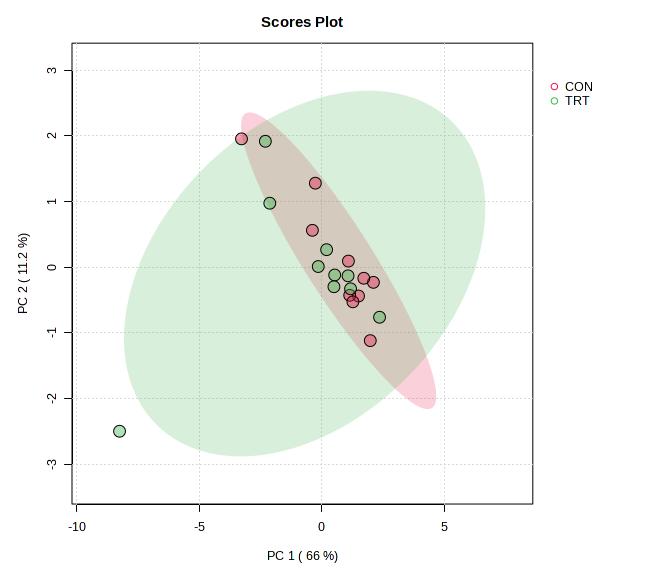


**F**

**C**

**Fig. S3** PCA scores plot for the comparison of volatile polar metabolite (panel **A**, **B**, and **C**) and fatty acid (panel **D**, **E**, and **F**) profiles of colostrum (panel **A** and **D**), transient milk (panel **B** and **E**), and mature milk (panel **C** and **F**) samples collected from sows fed a standard gestation diet from day 85 to 108 of gestation and standard lactation diet from day 109 of gestation until day 21 of lactation (**CON**; red color) or fed the CON diet top dressed with 1 g/sow/day of *E. gracilis*-derived β-glucan (**TRT**; green color).
